# Caring for Dying Parents in the Shadow of Childhood Maltreatment: Gendered Agency, Constraint, and Health

**DOI:** 10.1093/geroni/igaa057.1651

**Published:** 2020-12-16

**Authors:** Robin Tarter, Dena Hassouneh, Susan Rosenkranz

**Affiliations:** Oregon Health and Science University, Portland, Oregon, United States

## Abstract

Adult daughters represent the largest and fastest growing population of providers of unpaid care labor (UCL) to older adults with life limiting illness. Providing UCL to parents at the end of life is associated with significant and lasting risks of morbidity and mortality, especially for women with negative relationships with care recipients, and those who provide UCL based on constraining gendered expectations rather than agentic choice. While nearly one quarter of US women experience some form of maltreatment from parents during childhood, few studies have examined, or even acknowledged, the effect of trauma on the experience and health impact of family UCL. We used feminist poststructuralist informed dialogic narrative analysis to explore discursive constructions of agency and constraint in co-constructed life histories from 21 women who provided end of life UCL to older adult parents who maltreated them in childhood. For these women, parental childhood maltreatment influenced identity construction, social position, intersubjectivity, and vulnerability to victimization. For some, providing end-of-life UCL to the parents who maltreated them facilitated the mobilization of relational agency and identity validation. For others, providing UCL potentiated lifelong constraint, reinforcing their positions as non-agents and leading to significant psychical and emotional harm. End of life UCL for older adult parents represents a crucible out of which either healing or re-traumatization can arise. Our findings will be leveraged to inform clinical practice and policy to support the growing population women trauma survivors providing UCL to older adult parents, reducing negative outcomes for those at the greatest risk.

